# A Customized Pigmentation SNP Array Identifies a Novel SNP Associated with Melanoma Predisposition in the *SLC45A2* Gene

**DOI:** 10.1371/journal.pone.0019271

**Published:** 2011-04-29

**Authors:** Maider Ibarrola-Villava, Lara P. Fernandez, Santos Alonso, M. Dolores Boyano, Maria Peña-Chilet, Guillermo Pita, Jose A. Aviles, Matias Mayor, Cristina Gomez-Fernandez, Beatriz Casado, Manuel Martin-Gonzalez, Neskuts Izagirre, Concepcion De la Rua, Aintzane Asumendi, Gorka Perez-Yarza, Yoana Arroyo-Berdugo, Enrique Boldo, Rafael Lozoya, Arantxa Torrijos-Aguilar, Ana Pitarch, Gerard Pitarch, Jose M. Sanchez-Motilla, Francisca Valcuende-Cavero, Gloria Tomas-Cabedo, Gemma Perez-Pastor, Jose L. Diaz-Perez, Jesus Gardeazabal, Iñigo Martinez de Lizarduy, Ana Sanchez-Diez, Carlos Valdes, Angel Pizarro, Mariano Casado, Gregorio Carretero, Rafael Botella-Estrada, Eduardo Nagore, Pablo Lazaro, Ana Lluch, Javier Benitez, Conrado Martinez-Cadenas, Gloria Ribas

**Affiliations:** 1 Servicio de Oncologia Medica y Hematologia, Fundacion Hospital Clinico Universitario-INCLIVA, Valencia, Spain; 2 Programa Genetica Humana, CNIO, Madrid, Spain; 3 Department of Genetica, Antropologia Fisica y Fisiologia Animal, Universidad del Pais Vasco, Leioa, Spain; 4 Department of Biologia Celular e Histologia, Universidad del Pais Vasco, Leioa, Spain; 5 Department of Dermatologia, Hospital Gregorio Marañon, Madrid, Spain; 6 Department of Dermatologia, Hospital La Paz, Madrid, Spain; 7 Department of Dermatologia, Hospital Ramon y Cajal, Madrid, Spain; 8 Unidad de Cirugia Oncologica, Hospital Provincial Castellon, Castellon, Spain; 9 Servicio de Dermatologia, Hospital Provincial Castellon, Castellon, Spain; 10 Servicio de Dermatologia, Hospital General Castellon, Castellon, Spain; 11 Servicio de Dermatologia, Hospital La Plana, Vila-real, Castellon, Spain; 12 Servicio de Dermatologia, Hospital de Cruces, Baracaldo, Spain; 13 Servicio de Dermatologia, Hospital de Basurto, Bilbao, Spain; 14 Department of Dermatologia, Hospital Dr Negrin, Las Palmas de Gran Canaria, Spain; 15 Instituto Valenciano de Oncologia, Valencia, Spain; 16 Laboratorio de Biopatologia Molecular, Hospital Provincial Castellon, Castellon, Spain; Instituto de Ciencia de Materiales de Madrid - Instituto de Biomedicina de Valencia, Spain

## Abstract

As the incidence of Malignant Melanoma (MM) reflects an interaction between skin colour and UV exposure, variations in genes implicated in pigmentation and tanning response to UV may be associated with susceptibility to MM. In this study, 363 SNPs in 65 gene regions belonging to the pigmentation pathway have been successfully genotyped using a SNP array. Five hundred and ninety MM cases and 507 controls were analyzed in a discovery phase I. Ten candidate SNPs based on a p-*value* threshold of 0.01 were identified. Two of them, rs35414 (*SLC45A2*) and rs2069398 (*SILV/CKD2*), were statistically significant after conservative Bonferroni correction. The best six SNPs were further tested in an independent Spanish series (624 MM cases and 789 controls). A novel SNP located on the *SLC45A2* gene (rs35414) was found to be significantly associated with melanoma in both phase I and phase II (P<0.0001). None of the other five SNPs were replicated in this second phase of the study. However, three SNPs in *TYR*, *SILV/CDK2* and *ADAMTS20* genes (rs17793678, rs2069398 and rs1510521 respectively) had an overall p-*value*<0.05 when considering the whole DNA collection (1214 MM cases and 1296 controls). Both the *SLC45A2* and the *SILV/CDK2* variants behave as protective alleles, while the *TYR* and *ADAMTS20* variants seem to function as risk alleles. Cumulative effects were detected when these four variants were considered together. Furthermore, individuals carrying two or more mutations in *MC1R*, a well-known low penetrance melanoma-predisposing gene, had a decreased MM risk if concurrently bearing the *SLC45A2* protective variant. To our knowledge, this is the largest study on Spanish sporadic MM cases to date.

## Introduction

Malignant melanoma (MM) is a cancer of melanocytes (pigment-producing cells) located predominantly along the basal layer of the epidermis, but also found in mucous membranes. The most relevant epidemiologic characteristic of MM is its increasing incidence among Caucasian populations [1]. The aetiology of MM remains unclear but it is known that both genetic and environmental factors influence the development of sporadic disease [2].

If putative genetic pathways involved in MM susceptibility are considered, the human pigmentation pathway seems to have a main role in the pathogenesis of the disease. MM predisposition is highest in fair skin, high mole count, blond or red-haired individuals who never tan and always burn (Fitzpatrick's phototypes I and II) [2]. Since the incidence of MM reflects an interaction between skin colour and UV exposure, variations in genes implicated in pigmentation and tanning response to UV may be associated with susceptibility to MM and may be useful predictors of MM risk in the general population [3]–[6].

In recent years, considerable effort has been devoted to understanding the role of low penetrance genes in the pathogenesis of cancer. Linkage analysis in families affected with cancer has led to the identification of highly penetrant MM cancer genes: *CDKN2A* (MIM#60160) and *CDK4* (MIM#609048) [2]. However, individual high-risk alleles are generally uncommon, hence many other low penetrance genes have been considered to be involved in the pathogenesis of MM, each contributing a small effect to the total genetic component [7]. To date, only one gene, the melanocortin 1 receptor gene (*MC1R*; MIM#155555), is known to unequivocally account for a substantial variation in the incidence of sporadic MM [8]–[13]. Moreover, several other genes, such as *OCA2* (MIM#611409), *ASIP* (MIM#600201) and *SLC45A2* (MIM#606202) have also emerged as being potentially important in MM susceptibility [14]–[18]. Recently, with the emergence of “large-scale genome-wide association studies” (GWAS), it has become feasible to perform a systematic search for other low to moderate risk-conferring alleles that could contribute to sporadic MM. Several GWAS have identified genomic regions associated with MM or with pigmentation characteristics such as hair and eye colour, skin sensitivity to sun and freckling. Genetic regions near the *ASIP* (MIM#600201), *E2F1* (MIM#189971), *SLC24A4* (MIM#210750, *KITLG (MIM#*611664), *TYR* (MIM#606933), *OCA2/HERC2* (MIM#605837), *MC1R* (MIM#155555) or *SLC45A2* (MIM#606202) genes [14], [19]–[21] seem to be involved in pigmentation and/or MM susceptibility.

Considering the involvement of pigmentation in MM predisposition, we investigated the role of 384 polymorphisms in 65 pigmentation gene regions in MM susceptibility using two large independent series of Spanish samples (1214 MM cases and 1296 controls in total). To our knowledge, this is the largest study on Spanish sporadic MM cases to date.

## Results

From the initial list of 384 variants, 21 SNPs (5.4%) were discarded due to failed genotyping (no PCR amplification, insufficient intensity for cluster separation or poor cluster definition). Overall, 363 SNPs were studied, representing all initially selected genes. Evidence of departure from HWE among controls was observed for ten SNPs, although none of them remained statistically significant after a conservative Bonferroni correction for multiple testing. The number of SNPs successfully genotyped in each gene in discovery phase I is included in Table S1. Their genomic positions are summarized as follows: 256 intronic (66.7%), 47 5′ UTR upstream (12.5%), 64 3′ UTR downstream (16.7%) and 16 coding (4.2%).

### Comparison of allele frequencies

**Figure 1:**
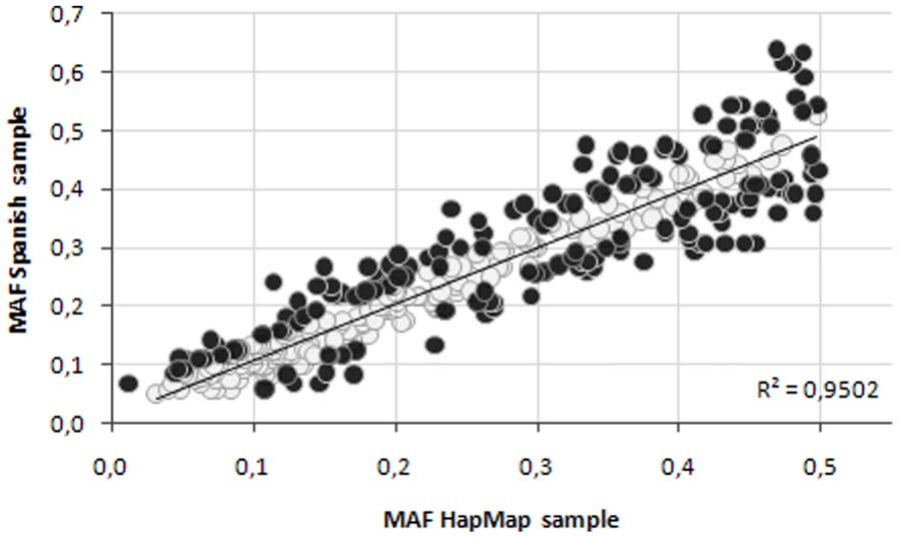
Comparison of minor allele frequencies, Spanish versus HapMap European data. Black dots represent values that significantly differ from HapMap European data, when sample size was considered (not corrected by Bonferroni method).

Estimated allele frequencies for all 363 SNPs based on the Spanish sample are provided in Table S1. We compared our Spanish frequencies to those of HapMap CEU subjects, using the HapMap minor allele as the reference (Figure 1). Frequencies were very similar, with a high positive correlation (R^2^) of 0.85. For 20 SNPs (5.5%), the minor allele reported in HapMap was found to be the most frequent allele in the Spanish sample (values above the dotted horizontal line in Figure 1). For 143 of the 363 SNPs considered (39%), one-sample t-test gave evidence that the corresponding Spanish minor allele frequency (MAF) differed from that published in HapMap (p<0.05) (shown in dark grey in Figure 1). However, when the Bonferroni correction was applied (p<0.00015), none of the SNPs seemed to be significantly different from HapMap data.

### Associations with MM risk

**Figure 2:**
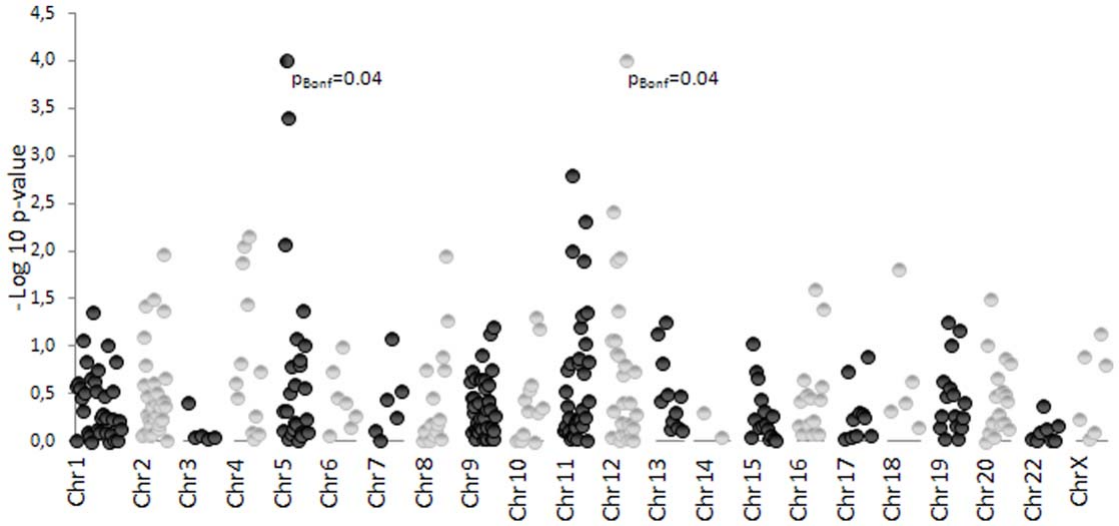
Pigmentation SNP array association results. The –log10 of the allelic p-*values* from 363 polymorphic SNPs comparing 590 melanoma cases and 507 controls of Spanish origin are represented. The chromosomal SNP distribution is shown. Two values remained statistically significant after Bonferroni correction “pBonf = 0.04”.

Representation of −log10 p-*values* for the comparison of MAFs between the 590 Spanish MM cases and the 507 controls are detailed in Figure 2. Considering a p-*value* threshold of 0.05, 30 SNPs located in 18 genes on eleven individual chromosomes were associated with MM in the Spanish population. Detailed information on rs number, genes, chromosome location, MAFs, OR, 95% CI and p-*values* for comparisons between cases and control subjects are represented in Table S2. However, if a more restrictive p-*value* threshold of 0.01 is established, only ten SNPs, located in six genes (one in each of *ADAMTS20*, *TYR* and *SILV-CDK2*; two in each of *KIT* and *MYO7A*; and three in *SLC45A2*) on six individual chromosomes constituted the top ten MM associated SNPs in our Spanish population. Among them, two SNPs (rs35414 and rs2069398), located in two different genes (*SLC45A2* and *SILV-CDK2* respectively), are statistically significant after applying the Bonferroni correction for multiple comparisons (p-*value* = 0.0001). Results, including SNP chromosome and gene region, allele frequencies of the top ten MM-associated SNPs, p-*values* from comparisons between cases and controls and p-*values* for Bonferroni correction are summarized in Table 1.

**Table 1:** Top ten SNPs associated with Malignant Melanoma p<0.01.

Gene			Ma/M	MAF	MAF			
Symbol	Chr	SNP	Allele	Cases	Controls	OR (95% CI)	p-*value*	p-B
*KIT*	4	rs759083	C/G	0.40	0.34	1.26 (1.06–1.50)	0.009	1
		rs13135792	T/C	0.38	0.33	1.27 (1.07–1.52)	0.0071	1
*SLC45A2*	5	rs35405	C/T	0.48	0.42	1.26 (1.06–1.49)	0.0084	1
		**rs35414**	**C/T**	**0.37**	**0.45**	**0.71 (0.60–0.84)**	**0.0001**	**0.04**
		rs35415	C/A	0.39	0.47	0.73 (0.62–0.87)	0.0004	0.14
*MYO7A*	11	rs948970	C/G	0.44	0.49	0.80 (0.68–0.94)	0.0098	1
		rs3758708	G/A	0.13	0.08	1.63 (1.20–2.22)	0.0016	0.58
*TYR*	11	rs17793678	C/T	0.28	0.23	1.32 (1.09–1.60)	0.0049	1
*ADAMTS20*	12	rs1510521	T/C	0.37	0.31	1.30 (1.09–1.55)	0.0039	1
*SILV-CDK2*	12	**rs2069398**	**G/A**	**0.06**	**0.10**	**0.52 (0.38–0.72)**	**0.0001**	**0.04**

Ma, Major; Mi, Minor; MAF, minor allele frequency; OR, odds ratio per minor allele; CI, confidence interval; B, Bonferroni.

Bold indicates statistically significant results.

**Figure 3:**
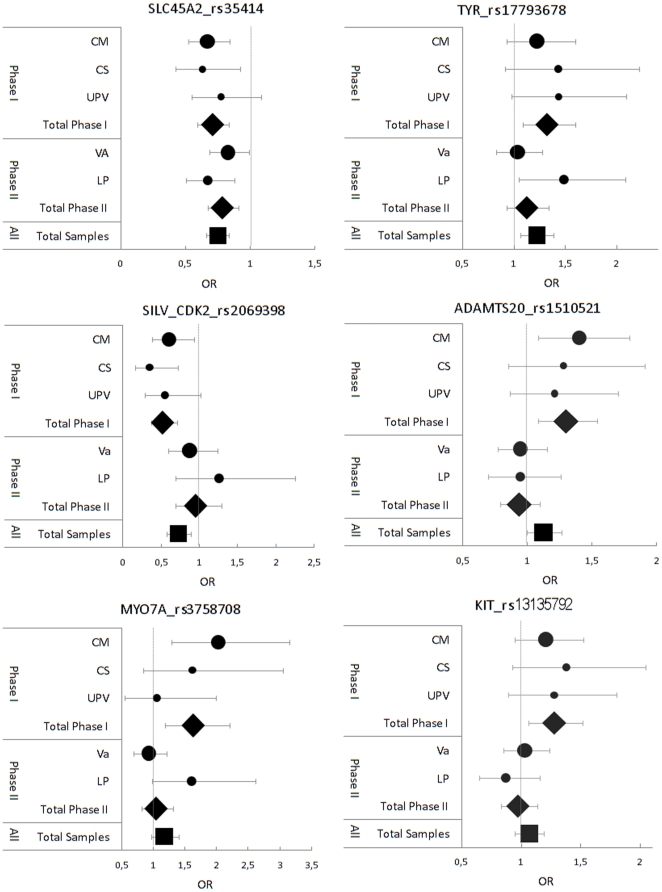
Forest plot showing the six most associated polymorphisms with melanoma risk predisposition. Dots represent odds ratios in the treated sub-groups versus those in control groups. Diamond shapes represent pooled results for each phase, discovery (phase I) and validation (phase II). Squares represent the overall odds ratios. The size of the square is proportional to the number of individuals, and error bars represent 95% confidence intervals.

From these ten associated SNPs, the one with the best p-*value* in each gene (six SNPs) was chosen to be studied in validation phase II, consisting on an independent Spanish set of 624 MM cases and 789 controls. The concordance rate between duplicate samples in the quality control analysis was 100%. None of the SNPs presented evidence of departure from HWE. One SNP, rs35414 in *SLC45A2* gene, had an unadjusted p-*value = *0.002 in phase II and an overall p-*value*<0.0001 with an overall OR = 0.75 (95% CI 0.67–0.84) (Figure 3). None of the other five SNPs tested in phase II reached statistical significance at this stage. However, three of them, located in *TYR*, *SILV-CDK2* and *ADAMTS20* had an overall p-*value*<0.05 when phase I and II were considered together (1214 MM cases and 1296 controls). These three SNPs were: rs17793678 in an intronic region of the *TYR* gene with an overall OR = 1.22 (95% CI 1.07–1.34, p-*value* = 0.003); rs2069398 located in exon 1 of the *CDK2* gene and a putative promoter region of the *SILV* gene, with an overall OR = 0.73 (95% CI 0.58–0.90, p-*value* = 0.004); and rs1510521 in an *ADAMTS20* intron with an overall OR = 1.13 (95% CI 1.001–1.27, p-*value* = 0.048). A fourth SNP, rs3758708 in the *MYO7A* gene, was marginally associated with MM (OR = 1.18, 95% CI 0.98–1.41, p-*value* = 0.09).

We observed some degree of epistatic protective interaction between rs35414 (*SLC45A2* gene) and rs2069398 (*SILV-CDK2* gene region) when considering rare allele carriers at both *loci*. Results including joint genotypes (rs35414 and rs2069398), status of individuals (controls or cases), total number of samples, ORs (95% CI) and p-*values* are shown in Table 2a. We observed a significant decrease in OR when two or three of the rare alleles were present at both *loci*, rs35414 C/T and rs2069390 A/-, OR 0.54 (95% CI 0.39–0.75) p-*value* = 0.0003, and when three or four rare alleles, rs35414 T/T and rs2069390 A/-, were present we obtained and OR = 0.31 (95% CI 0.18–0.55) with p-*value* = 0.0001.

**Table 2:** Interactions between protective (a) and risk (b) variants and their effect on MM susceptibility.

a						
Joint genotype		Spanish population				
rs35414 (*SLC45A2*)	rs2069398 (*SILV*)	Cases	Controls	N	OR (95%CI)	p-*value*
C/C	G/G	431	340	771	1	REF
	A/-	54	57	111	0.75 (0.50–1.11)	0.15
C/T	G/G	485	538	1023	**0.71 (0.59–0.86)**	**0.0004**
	A/-	70	103	173	**0.54 (0.39–0.75)**	**0.0003**
T/T	G/G	149	199	348	**0.59 (0.46–0.76)**	**0.0001**
	A/-	17	43	60	**0.31 (0.18–0.55)**	**0.0001**

b						
Joint genotype		Spanish population				
rs17793678 (*TYR*)	rs1510521 (*ADAMTS20*)	Cases	Controls	N	OR (95%CI)	p-*value*
C/C	A/A	297	371	668	1	REF
	G/-	361	382	743	1.18 (0.96–1.46)	0.122
C/T	A/A	203	224	427	1.13 (0.89–1.44)	0.321
	G/-	260	230	490	**1.41 (1.12–1.78)**	**0.004**
T/T	A/A	40	25	65	**1.99 (1.19–3.35)**	**0.009**
	G/-	42	34	76	1.54 (0.96–2.48)	0.088

c						
Joint genotype		Spanish population				
*MC1R*	rs35414 (*SLC45A2*)	Cases	Controls	N	OR (95%CI)	p-*value*
0	C/C	63	39	102	1	REF
	T/-	95	133	228	**0.44 (0.27–0.71)**	**0.001**
1	C/C	71	39	110	1.13 (0.65–1.97)	0.78
	T/-	119	114	233	0.65 (0.40–1.04)	0.08
2/+	C/C	45	6	51	**4.64 (1.85–11.58)**	**0.001**
	T/-	64	18	82	**2.20 (1.14–4.23)**	**0.02**

OR, odds ratio per minor allele; CI. Confidence interval; REF. Reference value.

Bold indicates statistically significant results.

Furthermore, some degree of epistatic risk interaction was also seen between rs17793678 (*TYR*) and rs1510521 (*ADAMTS20*) when considering rare alleles at both *loci*. Results including joint genotypes (rs17793678 and rs1510521), status of individuals (controls or cases), total number of samples and ORs (95% CI) and p-*values* are shown in Table 2b. We observed a risk effect when two or three rare alleles were present at both *loci*, rs17793678 C/T and rs1510521 G/-, OR = 1.41 (95% CI 1.12–1.78) p-*value* = 0.004, and when three or four rare alleles, rs17793678 T/T and rs1510521 G/- were present, we obtained an OR = 1.54 (95% CI 0.96–2.48) with a trend toward significance (p-*value* = 0.088).

The role of *SLC45A2* in melanoma predisposition was further analyzed in relation to *MC1R*, the main low penetrance gene associated to melanoma. Since both genes have been studied by our group, the interaction effects between both *loci* (see Table 2c) were investigated [22]. A great reduction of risk was detected when the rare protective alleles at *SLC45A2* were combined with two or more *MC1R* variants (OR = 2.20; 95% CI 1.14–4.23; p-*value* = 0.02), in comparison to individuals carrying only *MC1R* variants (OR = 4.64; 95% CI 1.85–11.58; p-*value* = 0.001). Individuals having only one *MC1R* mutation also show a reduction in MM risk, although this decline does not seem to reach significant values (Table 2c). These results confirm the protective role of the rs35414 variant in *SLC45A2* regarding MM risk. Additional comparisons with *HERC2/OCA2* rs12913838 did not add more information.

### Associations of top ten MM-associated SNPs with phenotypic characteristics

Evidence of association with phenotypic characteristics for the top ten MM-associated SNPs was also assessed. Both *KIT* SNPs rs759083 and rs13135792 appeared to be associated with both light hair colour (p = 0.0021 and p = 0.0072) and childhood sunburns (p = 0.0112 and p = 0.0167). *SLC45A2* SNPs rs35414 and rs35415 were associated with dark skin colour (p = 0.028 and p = 0.0485) and only rs35414 with dark hair colour (p = 0.0183). Two different SNPs in *MYO7A* were associated with dark hair colour (rs948970) (p = 0.04), and childhood sunburn (rs3758708) (p = 0.0474). One SNP in the *TYR* gene (rs17793678) was associated with light eye colour (p = 0.0239). Likewise, the *ADAMTS20* gene was associated with light eye colour (p = 0.0339), blond or red hair colour (p = 0.0353) and with number of naevi <50 (rs1510521; p = 0.0338). Finally, *SILV-CDK2* SNP rs2069398 was associated with absence of childhood sunburns (p = 0.0353). Table S3 summarizes results for eye, skin and hair colour, presence of childhood sunburns, number of naevi and the presence of solar lentigines. Results in bold represent those with associated p-*values*<0.05.

### Haplotype structure at the *SLC45A2* locus with reported associations with MM

The *SLC45A2* gene haplotype structure (obtained using HapMap data) is shown in Figure S1. This locus, located in chromosome 5, is represented by two main haploblocks, one located at the 3′ end of the gene (2 kb), and another one comprising the 5′ end of the gene (13 kb), which includes part of intron 1 and the closest putative promoter. SNPs located between these two LD blocks are not defined in any additional block structure, probably due to their low minor allele frequencies.

Additional analyses using HapMap data were performed. These analyses included three SNPs previously associated with MM at the *SLC45A2* locus: p.F374L (rs16891982), rs35391 and rs28777 [15], [23], [24], together with the rs35414 variant associated with MM in this study. The four SNPs under consideration are located in chromosome 5 in the following order: rs16891982 (p.F374L); rs35391; rs28777 and rs35414 (HapMap). The analysis revealed five different haplotypes (see Table S4), of which two are by far the most common: GCAC (66.7%) and GCA**T** (29.4%). This last haplotype carries the protective T allele at rs35414. Haplotype CCCT (1%) carries the rare C allele at the p.F374L amino acid change as well as the rare C allele at rs28777. Minor C allele at rs28777 is only present in a low frequency haplotype (GCCC 2%). In the HapMap database, we did not observe any haplotypes carrying the rare T allele at rs35391 due to its low frequency in Caucasians.

## Discussion

In this study, 363 tag SNPs in 65 pigmentation-related gene regions were successfully genotyped in 590 MM cases and 507 controls in discovery phase I. In addition, 624 MM cases and 789 controls were genotyped for the top six MM-associated SNPs in validation phase II. Thus, a total of 1214 MM cases and 1296 controls have been evaluated in this study. Among 363 SNPs in 65 pigmentation gene regions, allele frequencies were highly correlated between the HapMap (CEU) and the Spanish samples (R^2^ = 0.85). Despite of 143 SNPs with minor allele frequencies different from those published in HapMap, none remained significant after multiple testing (Bonferroni correction). Although the published HapMap MAFs should probably not be taken as definitive (at least for a Southern European population), overall, the HapMap CEU sample is representative of the Spanish population. Therefore, the HapMap data can be used in assessing the appropriateness of SNPs for inclusion in association studies even though we are analyzing genes with putative phenotypic implications.

From discovery phase I, p-*values* below 0.05 were obtained for 30 SNPs and, of these, ten final candidate SNPs, based on an arbitrary p-*value* threshold of 0.01, were identified. We were encouraged to follow a validation phase due to two SNPs (rs35414 and rs2069398) remaining statistically significant after Bonferroni correction. After testing six of these ten SNPs in an independent Spanish series with 624 MM cases and 789 controls, one SNP (rs35414) on an intronic region of the *SLC45A2* gene significantly associated with melanoma was confirmed (p<0.0001). Three additional SNPs in *TYR*, *SILV/CDK2* and *ADAMTS20* (rs17793678, rs2069398 and rs1510521 respectively) had an overall p-*value*<0.05 when considering the whole data set (1214 MM cases and 1296 controls). However, in validation phase II, data for these three SNPs did not reach statistical significant results *per se*.

Recent GWAS have unveiled association between SNPs or genetic variants in *MC1R*, *TPCN2*, *ASIP*, *KITLG*, *SLC24A5*, *TYR*, *IRF4*, *OCA2/HERC2*, *SLC24A4*, *SLC45A2*, *TYRP1*, and pigmentation as well as melanoma. These findings emphasize the contribution of pigmentation to melanoma predisposition and tumourigenesis through gene-environment interactions [19]–[21], [25].

The top predisposing MM-associated gene in our study has functions related to melanosomal formation, maturation and transportation. The *SLC45A2* gene (MIM#606202) encodes a protein that acts, presumably, as a membrane-associated transporter [26]–[28]. *SLC45A2* exhibits structural homology to plant sucrose-proton symporters and probably directs the traffic of melanosomal proteins and other substances to the melanosomes [29]. *SLC45A2* mutations cause pigmentation variation in several organisms [27], [30]–[33]. In humans, pathogenic mutations in *SLC45A2* lead to type IV oculocutaneous albinism (OCA4) [26]. *SLC45A2* mutations disrupt tyrosinase processing and trafficking at the post-Golgi level [34], [35]. Other variants located on the promoter and in exonic sequences on the *SLC45A2* gene have been shown to be significantly associated with dark hair, skin, and eye pigmentation in Caucasian populations [15], [23], [28], [36]. Previous results from our group and others show that the coding variant c.1122C>G (p.F374L, rs16891982) has been associated with protection against melanoma susceptibility in Southern European populations [15], [23]. In addition, a recent study by Duffy and cols. ([24]) has detected protective effects of *SLC45A2* through three SNPs in that gene: rs16891982, rs35391 and rs28777. This last study concludes that the two noncoding SNPs, rs35391 and rs28777, are in LD with each other and are not significant melanoma risk predictors in a multivariate logistic regression models after adjusting for rs16891982 (p.F374L). They confirm that rs16891982 is the SNP most strongly associated with melanoma risk in their population. These three SNPs are placed in the middle region of the *SLC45A2* gene, a segment of the gene that, according to HapMap data, does not belong to any block structure (probably due to their low minor allele frequencies in the Caucasian population where they were genotyped) (Figure S1).

The results obtained in this study support the protective role of *SLC45A2* and propose two novel intronic SNPs associated with MM predisposition. Both of these SNPs, rs35414 and rs35415, are located inside intron 1 of the *SLC45A2* gene. rs35415 (p-*value* 0.0004 in phase I) was selected as the tag of the LD block located at the 5′ end of the gene, while rs35414, studied in phase I and validated in phase II (global p-*value* = 0.0001), is located in a region without apparent LD block structure (Figure S1). However, both SNPs (rs35414 and rs35415) are in strong linkage disequilibrium with each other (R^2^ = 0.95) (Figure S1). Both constitute the second most common haplotype of the gene (43%), also conferring protection against melanoma (p-*value* = 0.0001). Thus, the association of this 5′ end region to MM predisposition suggests a putative involvement of unknown functional variants, in LD with these SNPs, involving regulatory elements in the promoter region of the *SLC45A2* gene.

Furthermore, the protective role of the rs35414 variant in *SLC45A2* is reinforced due to its capability to modulate the MM risk conferred by the *MC1R* locus.

Although no additional SNPs were replicated in the second phase of the study, three of them had statistical significance when the entire data collection was taken as a whole. Therefore, we consider them as quite relevant for further validations in other populations.

SILV (MIM#155550) melanosomal matrix protein represents a melanoma specific antigen recognized by tumour infiltrating cytotoxic T lymphocytes [37]. It is also known as PMEL17, GP100 or ME20 [38] and it is crucial for proper formation and maturation of melanosomes. In stage II melanosomes, processed SILV protein aggregates to form fibrils, to which presumably the eumelanin pigment is attached [39]. It was found to be orthologous to the mouse *silver* locus. In this study, SNP rs2069398 was selected in order to represent the 5′end of the *SILV* gene. Interestingly, the *SILV* locus is flanked quite closely on each side by *CDK2* and *DGKA* at distances of 829 and 193bp, respectively, placed head to head with *CDK2* in such a way as to allow sharing or overlapping promoter elements [40]. rs2069398 is located in exon1 of the *CDK2* gene, resulting in the synonymous polymorphism c.84G>A, p.Glu28Glu. The significance of this gene arrangement and the putative functionality of this SNP are yet to be tested.

The human tyrosinase gene (*TYR*, MIM#606933) is another melanosomal membrane-bound enzyme that catalyzes the first two rate-limiting steps in the melanin biosynthesis pathway [41]. Mutations in the *TYR* gene cause the most severe form of oculocutaneous albinism OCA1 (*TYR*, MIM 606933, OCA1, MIM 203100) [42]. MM associated SNP rs17793678 is located in intron 1 and was selected as the tag for one of the five most frequent haplotypes formed at the promoter LD block in this gene (19 kb). Interestingly, *TYR* has previously been associated with melanoma in GWAS studies [18] performed in Caucasian populations other than our South European sample.

Finally, the ADAMTS20 protein (A disintegrin-like and metalloprotease domain with thrombospondin type-1 motifs, MIM#611681) is a member of a family of secreted metalloproteases that can process a variety of extracellular matrix components and secreted molecules. *Adamts20* mutations in *belted* (*bt*) mice cause white spotting of the dorsal and ventral torso, indicative of defective neural crest-derived melanoblast development and thus implicating metalloproteases in skin pigmentation [43]. A role for *Adamts20* in melanoblast survival has been described [44]. In our study, SNP rs1510521 was selected as the tag of the most frequent haplotype of the large LD block at the 3′ end of the gene (107 kb), containing the majority of the gene region with the exception of the first three exons. Additional studies are needed in order to clarify the role of *ADAMTS20* in MM susceptibility.

Since *SLC45A2* and *TYR* genes have already been associated with MM [15], [18], [23], [24], it seemed biologically plausible that genetic interactions would be detected between protective *SLC45A2* and *SILV/CDK2* variants and, similarly, between risks variants within *TYR* and *ADAMTS20*. Indeed, both effects were observed when summing up rare alleles as it has been described for other interactions among pigmentation genes [24], [45]. On one hand, a great reduction of risk was detected when rare alleles at *SLC45A2* and *SILV/CDK2* genes were combined (OR = 0.31; p = 0.0001). On the other hand, an increased risk, although marginally significant, appeared with the combination of rare alleles at *TYR* and *ADAMTS20* (OR = 1.54; p = 0.088) (See Table 2).

We were not able to clearly detect any other gene association with MM in our study. However, several candidate genes that may be of interest to other pigmentation and/or MM studies are present in the list of the 30 SNPs with p<0.05. Unfortunately, some genes already known to be associated with pigmentation were not selected due to reasons such as large gene size, lack of suitable tag SNPs within the gene, genes lying in high copy number regions, etc. We acknowledge the fact that our sample set comes from different hospitals distributed throughout different Spanish provinces. However, a recent work by Laayouni and cols. using a 300K-SNP Illumina array demonstrates lack of genetic heterogeneity within different Spanish regions including the Basque country [46].

In summary, we conducted a two-stage MM susceptibility case-control study (candidate SNP selection and replication). First, we screened 363 tag-SNPs in 65 pigmentation-related gene regions in a large Spanish case-control series. Then we validated observed associations in an independent Spanish series, making this the largest sporadic MM susceptibility study in the Spanish population up to now. One SNP (rs35414) on an intronic region of the *SLC45A2* gene was associated with MM risk after adjustment for multiple testing in phase I and II, indicating that common variation in *SLC45A2* is associated with protection from the disease. Nevertheless, further studies are welcomed to add weight to our conclusions. We also show that carrying this protective variant decreases the risk of developing MM in bearers of two or more mutations in *MC1R*, a well-known low penetrance MM predisposing gene. Moreover, three additional variants in *TYR*, *SILV/CDK2* and *ADAMTS20* (rs17793678, rs2069398 and rs1510521 respectively) had an overall p-*value*<0.05, emphasizing the importance of pigmentation genes on MM risk.

## Materials and Methods

### Study Subjects and Data Collection

#### Discovery Phase I

A total number of 590 sporadic MM cases were recruited from September 2004 to November 2008 at the Departments of Dermatology of five Spanish hospitals: Gregorio Marañon General University Hospital (Madrid), La Paz University Hospital (Madrid), Ramon y Cajal University Hospital (Madrid), Castellon Province Hospital (Castellon), Cruces University Public Hospital (Barakaldo) and Basurto University Public Hospital (Bilbao). Similarly, 507 cancer-free controls were recruited from the Madrid College of Lawyers, Gregorio Marañon Hospital, Castellon Province Hospital and the University of the Basque Country.

A standardized questionnaire was used to collect information on pigmentation characteristics (eye colour, hair colour, skin colour, number of naevi and presence of solar lentigines), the presence of childhood sunburns, Fitzpatrick's skin type classification (see Table S5), and personal and family history of cancer.

#### Validation Phase II

A second phase of the study, consisting of an independent validation series, was composed of 624 non-related sporadic MM cases obtained from the Departments of Dermatology of Instituto Valenciano de Oncologia (Valencia) and Hospital Dr. Negrin from Las Palmas (Gran Canaria), Spain. A total of 789 cancer-free controls from the same two institutions were recruited. All subjects came from the geographical regions covered by the hospitals involved in the study. A recent work by Laayouni and cols. demonstrates lack of genetic heterogeneity within different Spanish regions, including the Basque country, by genome-wide single nucleotide polymorphism (SNP) array [46]. Sample distribution is listed in Table 3 (a+b).

**Table 3:** Sample distribution along discovery (phase I) and validation (phase II) sets*.

Discovery Group (Phase I)			
Sub-Group	Institution Origin	Cases (N = 590)	Controls (N = 507)
		n (%)	n (%)
**CM**	Gregorio Marañon Hospital	155 (26.27)	27 (5.33)
	Ramon y Cajal Hospital	120 (20.34)	0 (0.00)
	La Paz Hospital	72 (12.2)	0 (0.00)
	Madrid College of Lawyers	0 (0.00)	218 (43.00)
	**Sub-Total**	**347 (58.81)**	**245 (48.32)**
**CS**	Castellon Province Hospital	110 (18.64)	104 (20.51)
	**Sub-total**	**110 (18.64)**	**104 (20.51)**
**PV**	Basurto Hospital	110 (18.64)	0 (0.00)
	Cruces Hospital	23 (3.90)	0 (0.00)
	University of the Basque Country	0 (0.00)	158 (31.16)
	**Sub-Total**	**133 (22.54)**	**158 (31.16)**

Validation Group (Phase II)			
Sub-Group	Institution Origin	Cases (N = 624)	Controls (N = 507)
		n (%)	n (%)
**Va**	Instituto Valenciano Oncologia	412 (66.02)	558 (70.72)
**LP**	Dr. Negrin Hospital	212 (33.97)	231 (29.27)

CM (Comunidad Madrid); CS (Castellon); PV (Pais Vasco); Va (Valencia); LP (Las Palmas de Gran Canaria).

*Total Discovery and validation sets were formed by 1214 Melanomas and 1296 control samples.

Genomic DNA from cases and controls was isolated from peripheral blood lymphocytes and diluted to a final solution of 50 ng/µl. For the control subjects, this was done using the MagNA Pure LC Instrument according to the manufacturer's protocol (Roche Applied Science, Mannheim, Germany) or the DNAzol procedure (Invitrogen, Eugene, OR, USA). The traditional saline method was used for MM cases. Quality control was performed for all samples before processing them on the Illumina arrays. DNA concentration was quantified in samples prior to genotyping by using Quant-iT PicoGreen dsDNA Reagent (Invitrogen, Eugene, OR, USA).

All subjects gave written informed consent and the study was approved by the Ethics Committee of Gregorio Marañon Hospital and University Clinic Hospital.

### Gene and SNP Selection

Previous literature, information of public databases and information based on expression arrays [47] were used to perform gene selection in our candidate gene approach study. Genes were classified using Gene Ontology categories [48] with the help of the DAVID programmes [49]. Finally, 65 gene regions were included in the study (final list of genes available in Table S6). They covered a broad range of biological processes. Nevertheless, the most overrepresented categories were pigmentation GO:0043473 (27 genes, p-*value* = 5.77×10^−32^); pigmentation during development GO:0048066 (19 genes with p-*value* = 2.13×10^−21^) and cellular pigmentation GO:0033059 (11 genes, p-*value* = 2.9×10^−18^). Another overrepresented category was membrane-bounded vesicle GO: 0031988 (12 genes, p-*value* = 1.39×10^−11^), that includes genes coding for proteins located in the melanosome membrane (specialized lysosome implicated in melanin transport). Thus, their functions are related to pigmentation.

Our group had already analyzed exonic and putative functional SNPs in 15 genes associated with pigmentation syndromes or associated to defects in mice pigmentation [50]. However, in the current study, selected genes were analyzed using tag-SNPs ranging from the hypothetical promoter area (approximately 10 kb upstream) until approximately 5 kb downstream of the gene. SNP codes, locations, and frequencies were obtained from the NCBI (www.ncbi.nlm.nih.gov/SNP), HapMap (www.hapmap.org) and Illumina databases. Finally, 384 tag-SNPs were chosen to infer Linkage Disequilibrium (LD) blocks according to the HapMap project. To ensure a high genotyping success rate, a minor allele frequency threshold of 0.05 in the HapMap CEU population and an ‘Illumina score’ of not less than 0.6 (as recommended by the manufacturer) were established in the SNP selection process.

### SNP Genotyping

SNPs were genotyped using the GoldenGate Genotyping Assay system according to the manufacturer's protocol (Illumina, San Diego, CA, USA). Genotyping was carried out using 350 nanograms of DNA per reaction. Genotyping specificity was assessed by including three DNA duplicates (two intra-assays and one inter-assay) and a negative control in each 96-well plate genotyped, yielding 100% consistent replication results. In addition, cases and control samples were always included in the same run. Genotypes were called using the proprietary software supplied by Illumina (BeadStudio, version 3.1.3.).

Validation set genotyping was carried out using the TaqMan platform according to the manufacturer's protocol (Applied Biosystems, Foster City, CA, USA). For this validation, the six selected SNPs were analyzed using Taqman^R^ SNP Genotyping Assays *TYR* (NM_000372.4) rs17793678, C_34306017_10; *KIT* (NM_000222.2) rs13135792, C_3288591_10; *SLC45A2* (NM_016180.3) rs35414, C_2390581_10; *SILV/CDK2* (NM_006928.3) rs2069398, C_15793372_10; *ADAMTS20* (NM_025003.3) rs1510521, C_7519176_10 and *MYO7A* (NM_000260.3) rs3758708, C_27475322_10. Fifteen nanograms of DNA were used for the genotyping reactions. The genotype of each sample was automatically determined by measuring allele-specific final fluorescence in an ABI Prism 7900HT Detection System, using SDS 2.3 software for allele discrimination (Applied Biosystems, Foster City, CA, USA). As a quality control measure, at least two sample duplicates and a non-template sample per 96-well plate were included. Genotypes were scored by two different personnel in the laboratory. A concordance rate of 100% was obtained for all the SNPs tested.

### Haplotype analysis

Haplotype information was obtained from the HapMap database (www.hapmap.org), using available data from the *SLC45A2* gene (Chromosome 5; 33,975,000–34,025,000; version 28).

### Statistical Analysis

Quality control processes and allelic and genotypic association tests were performed using the SNPator package in discovery phase I (www.snpator.com). Analyses performed in the validation set were conducted using SPSS v17.

For all polymorphisms studied, Fisher's exact test was used both to test for deviations from Hardy-Weinberg equilibrium (HWE) among controls and to compare allele counts between cases and controls. Correction for multiple testing was carried out using the Bonferroni method. Genotype-related odds ratios, their corresponding 95% confidence intervals and associated p-*values* were estimated via unconditional logistic regression. Multivariate logistic regression was also applied, including age (categorical: <30, 30–39, 40–49, 50–59, 60–69, 70–79, ≥80), sex (male, female), hair colour (brown/black, blond/red), skin colour (fair, brown), lentigines (yes, no) and childhood sunburn (yes, no) as covariates. Associations between the genotyped pigmentation genes and various pigmentation characteristics and sun sensibility were assessed via logistic regression. This was done for cases and controls combined, with eye colour (blue/green *versus* brown), hair colour (brown/black *versus* blond/red), skin colour (fair *versus* brown), number of naevi (≥25 *versus* <25), presence of lentigines (yes *versus* no) and childhood sunburn (yes *versus* no) as the outcome variables.

A Bonferroni-adjusted nominal p-*value* threshold of 0.00015 was used to account for multiple testing, based on 341 effective independent marker loci (N*) among 363 SNPs studied. N* was calculated by applying the web-based programme SNPSpD [51] to SNPs on individual chromosomes (http://gump.qimr.edu.au/general/daleN/SNPSpD/). Fisher's exact test had been used to account for differences in allele frequencies between HapMap CEU data and Spanish population data.

To study combinations of protective and risk genotypes, we used a 2×2 contingency table and a t-student test between *SLC45A2* and *SILV* (rs35414 and rs2069398 respectively), between *TYR* and *ADAMTS20* (rs17793678 and rs1510521 respectively) and between previous published results of *MC1R* (0, 1 or 2 variants) [22] and *SLC45A2* (rs35414).

Haplotype analysis was performed using the SNPstats programme available at: http://bioinfo.iconcologia.net/index.php?module=Snpstats.

## Supporting Information

Figure S1 LD map of the *SLC45A2* gene and tags selected in the study.
(DOC)

Table S1 List of 363 successfully genotyped SNPs, Spanish MAF, HapMap_CEU MAF, and HWE p-*value*.
(DOC)

Table S2 List of SNPs associated with Malignant Melanoma with p-*value*<0.05.
(DOC)

Table S3 Association between SNPs and phenotypic characteristics.
(DOC)

Table S4 Haplotype analysis of melanoma-associated variants previously reported in *SLC45A2*, including the candidate polymorphism detected in this study (rs35414).
(DOC)

Table S5 Classification of the Spanish samples (phase I) by age and sex.
(DOC)

Table S6 General information about genes, chromosomal location, SNPs per gene, associated disease, protein encoded and function in pigmentation.
(DOC)
