# Supplementary figures and images for: A Customized Pigmentation SNP Array Identifies a Novel SNP Associated with Melanoma Predisposition in the SLC45A2 Gene
Source: PLoS One. 2011 Apr 29;6(4):e19271. doi: 10.1371/journal.pone.0019271 (PMC3084811; doi:10.1371/journal.pone.0019271)

Figure S1. LD Map of the *SLC45A2* gene and tags SNPs selected in the study.


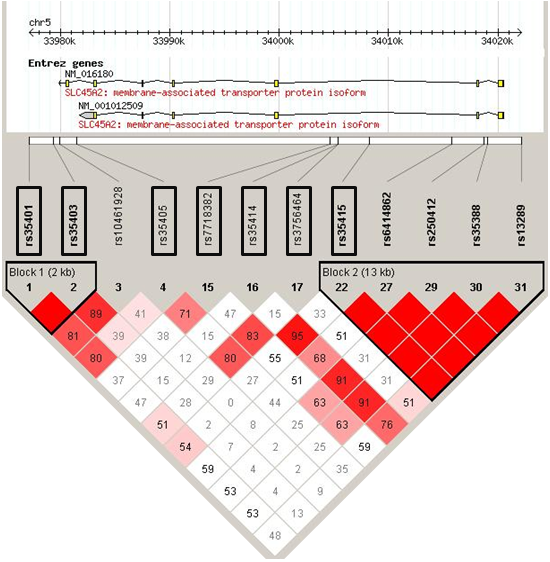

Supplement: Figure S1 — LD map of the SLC45A2 gene and tags selected in the study. (DOC) [file pone.0019271.s001.doc]
