# Supplementary material for: A Customized Pigmentation SNP Array Identifies a Novel SNP Associated with Melanoma Predisposition in the SLC45A2 Gene
Source: PLoS One. 2011 Apr 29;6(4):e19271. doi: 10.1371/journal.pone.0019271 (PMC3084811; doi:10.1371/journal.pone.0019271)
Supplement: Table S4 — Haplotype analysis of melanoma-associated variants previously reported in SLC45A2, including the candidate polymorphism detected in this study (rs35414). (DOC) [file pone.0019271.s005.doc]

| Table S4. Haplotype analysis of melanoma-associated variants previously reported in *SLC45A2*, including the candidate polymorphism detected in this study (rs35414) | | | | |
| --- | --- | --- | --- | --- |
| rs16891982  p.Phe374Leu  33,951,693^a,b^ | rs35391  intron 3  33,955,423^a,c^ | rs28777  intron 3  33,958,709^a,c^ | rs35414 intron 1  33,969,378^a,d^ | HapMap Frequency (%) |
| G | C | A | C | 66.7 |
| G | C | A | **T** | 29.4 |
| G | C | **C** | C | 2.00 |
| **C** | C | A | **T** | 1.00 |
| **C** | C | **C** | **T** | 1.00 |
| C : 0.017 | T : 0.004 | C : 0.022 | T : 0.340 | CEU* |
| ^a^ Localization according to NCBI hg19 chromosome 5.  ^b^ SNP analyzed in Fernandez et al. 2008; Guedj et al., 2008 and Duffy et al., 2010.  ^c^ SNP analyzed in Duffy et al., 2010.  ^d^ SNP analyzed in the current study.  *HapMap (version 28) minor allele frequency in Caucasians.  Bold indicates MM-associated allele. | | | | |
