# Supplementary material for: A Customized Pigmentation SNP Array Identifies a Novel SNP Associated with Melanoma Predisposition in the SLC45A2 Gene
Source: PLoS One. 2011 Apr 29;6(4):e19271. doi: 10.1371/journal.pone.0019271 (PMC3084811; doi:10.1371/journal.pone.0019271)
Supplement: Supplementary file 3 [file pone.0019271.s002.doc]

Table S1. List of 363 successfully genotyped SNPs, Spanish MAF, HapMap_CEU MAF and HWE p-value

**Gene**	**rs#**	**HM_CEU MAF**	**HM Allele**	**SP MAF Controls**	**SP Allele**	**HWE p-*value***
ADAM17	rs17524425	0.225	G	0.200	G	0.836
ADAM17	rs12473402	0.425	C	0.378	C	0.872
ADAM17	rs12475630	0.275	T	0.336	T	0.487
ADAM17	rs12992105	0.169	C	0.161	C	0.704
ADAM17	rs4258773	0.492	A	0.464	G	0.516
ADAMTS20	rs3764467	0.125	T	0.105	T	0.724
ADAMTS20	rs7297057	0.175	T	0.186	T	0.211
ADAMTS20	rs1510522	0.308	A	0.358	A	0.982
ADAMTS20	rs1510521	0.392	C	0.311	C	0.362
ADAMTS20	rs7960952	0.458	C	0.400	C	0.972
ADAMTS20	rs2048348	0.092	A	0.093	A	0.741
ADAMTS20	rs17093450	0.133	A	0.140	A	0.974
ADAMTS20	rs9634256	0.383	G	0.450	G	0.232
ADAMTS20	rs275630	0.364	A	0.283	A	0.633
ADAMTS20	rs2062731	0.125	G	0.108	G	0.842
AP3B1= HPS2	rs4703747	0.208	G	0.201	G	0.632
AP3B1= HPS2	rs10514134	0.100	A	0.091	A	0.964
AP3B1= HPS2	rs11746090	0.267	A	0.232	A	0.837
AP3B1= HPS2	rs34436	0.125	G	0.106	G	0.879
AP3B1= HPS2	rs389110	0.200	A	0.229	A	0.920
AP3B1= HPS2	rs2636986	0.200	A	0.229	A	0.920
AP3B1= HPS2	rs17191796	0.095	G	0.107	G	0.861
AP3B1= HPS2	rs6453373	0.075	A	0.073	A	0.553
AP3B1= HPS2	rs11742673	0.380	A	0.412	A	0.122
AP3B1= HPS2	rs12657894	0.383	A	0.384	A	0.899
AP3B1= HPS2	rs6453374	0.267	A	0.272	A	0.794
AP3B1= HPS2	rs13172957	0.259	G	0.335	G	0.661
AP3B1= HPS2	rs10805919	0.267	C	0.239	C	0.273
AP3D1	rs10413398	0.172	C	0.204	C	0.343
AP3D1	rs17604954	0.150	A	0.122	A	0.562
AP3D1	rs2240655	0.133	T	0.141	T	0.304
AP3D1	rs2238599	0.483	T	0.460	C	0.712
AP3D1	rs3786971	0.408	T	0.449	T	0.366
AP3D1	rs7256735	0.100	G	0.099	G	0.654
AP3M2	rs7823824	0.417	A	0.432	A	0.444
AP3M2	rs7009632	0.383	G	0.419	G	0.164
AP3M2	rs8178890	0.075	A	0.073	A	0.940
AP3M2	rs2070713	0.392	A	0.417	A	0.656
ASIP	rs6142129	0.275	G	0.331	G	0.272
ASIP	rs819133	0.117	T	0.143	T	0.574
BCL2	rs4987853	0.225	G	0.263	G	0.142
BCL2	rs4987852	0.083	G	0.054	G	0.660
BCL2	rs1564483	0.242	A	0.244	A	0.819
BCL2	rs949037	0.383	T	0.450	T	0.655
BCL2	rs1462129	0.400	T	0.471	T	0.711

BCL2A1	rs11636338	0.350	C	0.330	C	0.299
BCL2A1	rs17215263	0.271	G	0.315	G	0.894
BCL2A1	rs6495460	0.067	C	0.128	C	0.887
BLOC1S3=HPS7	rs7253652	0.125	C	0.091	C	0.836
CDH1	rs2902186	0.108	C	0.094	C	0.924
CDH1	rs16260	0.258	A	0.306	A	0.865
CDH1	rs11075699	0.473	A	0.417	G	0.533
CDH1	rs7188750	0.167	A	0.148	A	0.400
CDH1	rs8059139	0.058	G	0.048	G	0.802
CDH1	rs1801552	0.408	T	0.367	T	0.676
CDH1	rs8061932	0.158	C	0.166	C	0.430
CDH1	rs7203904	0.250	C	0.207	C	0.613
CDH1	rs2276329	0.085	G	0.045	G	0.524
CDH3	rs11075692	0.425	G	0.374	G	0.976
CDH3	rs1886700	0.158	A	0.121	A	0.723
CDH3	rs1124770	0.086	C	0.152	C	0.746
CDH3	rs3114398	0.217	G	0.296	G	0.946
CDH3	rs3118230	0.125	A	0.086	A	0.854
CDK4	rs2069502	0.367	A	0.239	A	0.990
CDK4	rs2270777	0.362	A	0.468	G	0.373
CDKN2A	rs3731257	0.267	T	0.295	T	0.892
CDKN2A	rs11515	0.125	G	0.172	G	0.426
CDKN2A	rs2518719	0.150	G	0.165	G	0.522
CDKN2A	rs3731239	0.375	C	0.320	C	0.302
CDKN2B	rs3218020	0.358	T	0.428	T	0.910
CDKN2B	rs2811712	0.108	G	0.113	G	0.566
CDKN2B	rs3218009	0.100	C	0.083	C	0.538
CDKN2B	rs1063192	0.400	C	0.374	C	0.398
CDKN2B	rs495490	0.125	C	0.117	C	0.493
CNO	rs10937751	0.292	A	0.274	A	0.587
CNO	rs3172604	0.392	G	0.345	G	0.930
CNO	rs4689527	0.375	G	0.350	G	0.273
CTNNBIP1	rs1220392	0.267	T	0.150	T	0.579
CTNNBIP1	rs2379107	0.172	G	0.177	G	0.594
CTNNBIP1	rs12128766	0.458	T	0.436	C	0.393
CTNNBIP1_LZIC	rs4846104	0.059	G	0.070	G	0.354
CTNNBIP1_LZIC	rs11828	0.233	G	0.196	G	0.487
DCT	rs9524493	0.450	G	0.464	G	0.074
DCT	rs3782973	0.142	C	0.170	C	0.198
DCT	rs3782972	0.136	G	0.171	G	0.218
DCT	rs9301959	0.483	C	0.447	C	0.357
DCT	rs9516418	0.408	C	0.363	C	0.566
DCT	rs2031527	0.192	T	0.191	T	0.639
EDNRB	rs11149080	0.407	G	0.403	G	0.554
EDNRB	rs4885491	0.117	A	0.153	A	0.975
EDNRB	rs3818416	0.208	T	0.266	T	0.830
EDNRB	rs3027110	0.075	C	0.083	C	0.900
EDNRB	rs3027129	0.085	T	0.065	T	0.753

F2RL1	rs639342	0.183	A	0.174	A	0.592
F2RL1	rs2242991	0.083	G	0.171	G	0.147
F2RL1	rs2243004	0.217	G	0.172	G	0.850
F2RL1	rs2243010	0.233	T	0.155	T	0.663
F2RL1	rs2243051	0.458	A	0.444	G	0.148
GNA11	rs4806907	0.350	C	0.381	C	0.660
GNA11	rs308039	0.192	T	0.190	T	0.832
GNA11	rs1104737	0.371	A	0.396	A	0.302
GNA11	rs2238625	0.492	T	0.448	C	0.552
GNA11	rs308054	0.358	A	0.424	A	0.135
GNA11	rs3786947	0.467	T	0.389	T	0.285
GNA11	rs404632	0.127	T	0.135	T	0.567
GNA11	rs3746069	0.142	A	0.157	A	0.751
GNA11	rs10407783	0.423	T	0.351	T	0.343
GNAQ	rs3858119	0.350	G	0.299	G	0.756
GNAQ	rs3780302	0.133	T	0.116	T	0.529
GNAQ	rs10869977	0.442	A	0.442	A	0.925
GNAQ	rs17724885	0.108	G	0.062	G	0.760
GNAQ	rs1328534	0.225	A	0.253	A	0.491
GNAQ	rs1328533	0.392	C	0.342	C	0.453
GNAQ	rs12686139	0.464	C	0.458	T	0.296
GNAQ	rs4745672	0.433	T	0.498	T	0.859
GNAQ	rs10781468	0.250	T	0.267	T	0.071
GNAQ	rs1410552	0.300	A	0.300	A	0.673
GNAQ	rs2296937	0.125	G	0.114	G	0.734
GNAQ	rs7028873	0.300	T	0.297	T	0.560
GNAQ	rs4237275	0.317	C	0.236	C	0.839
GNAQ	rs17786974	0.133	T	0.074	T	0.752
GNAQ	rs11145647	0.195	C	0.201	C	0.933
GNAS	rs965808	0.275	C	0.260	C	0.739
GNAS	rs6064714	0.133	G	0.132	G	0.383
GNAS	rs6123832	0.450	T	0.433	T	0.718
GNAS	rs6026561	0.425	C	0.400	C	0.498
GNAS	rs2145288	0.340	G	0.304	G	0.839
GNAS	rs12625436	0.308	A	0.419	A	0.975
GNAS	rs6026574	0.317	T	0.293	T	0.309
GNAS	rs6092704	0.125	C	0.089	C	0.628
GNAS	rs13831	0.258	T	0.239	T	0.432
GNAS	rs6100269	0.102	T	0.100	T	0.993
GNAS	rs919197	0.392	T	0.478	T	0.564
GNAS	rs234627	0.325	T	0.262	T	0.871
GNAS	rs234623	0.440	G	0.494	G	0.029 *
GPR143	rs5979160	0.222	C	0.163	C	0.000 ***
GPR143	rs2521578	0.083	A	0.123	A	0.000 ***
GPR143	rs6654731	0.111	C	0.048	C	0.002 ***
GPR143	rs11095519	0.122	A	0.139	A	0.000 ***
GPR143	rs2732872	0.267	C	0.181	C	0.000 ***
GPR143	rs2521667	0.222	G	0.155	G	0.000 ***

HPS1	rs3750605	0.225	T	0.202	T	0.301
HPS1	rs1739	0.333	C	0.315	C	0.020 *
HPS1	rs7921146	0.217	A	0.209	A	0.348
HPS1	rs10883094	0.267	A	0.210	A	0.603
HPS1	rs12242431	0.150	C	0.107	C	0.957
HPS1	rs1061135	0.392	T	0.496	T	0.860
HPS1	rs1886728	0.408	C	0.456	C	0.200
HPS1	rs17535384	0.208	G	0.131	G	0.878
HPS1	rs10786422	0.292	T	0.276	T	0.780
HPS1	rs7075080	0.379	G	0.430	G	0.788
HPS4	rs1894707	0.458	C	0.371	C	0.819
HPS4	rs3213583	0.083	A	0.108	A	0.607
HPS4	rs16982145	0.058	C	0.039	C	0.791
HPS4	rs3747129	0.175	A	0.152	A	0.255
HPS4	rs17401652	0.117	T	0.102	T	0.608
HPS4	rs9608491	0.250	G	0.187	G	0.794
HPS4	rs9613187	0.133	T	0.112	T	0.565
HPS5	rs12218	0.386	A	0.480	G	0.289
HPS5	rs7131332	0.267	G	0.329	G	0.266
HPS5	rs2049129	0.175	G	0.163	G	0.494
HPS5	rs4757637	0.292	C	0.358	C	0.313
HPS5	rs2305564	0.367	T	0.487	A	0.733
HPS5	rs4353250	0.258	T	0.294	T	0.641
HPS6	rs6584475	0.347	C	0.373	C	0.461
HPS6	rs3737243	0.142	A	0.115	A	0.564
HPS6	rs3816	0.133	G	0.228	G	0.257
HRK	rs10507275	0.158	A	0.157	A	0.364
HRK	rs4767462	0.079	G	0.063	G	0.994
HRK	rs1112700	0.158	T	0.140	T	0.283
HRK	rs884378	0.267	T	0.246	T	0.254
HTR2B	rs2161891	0.267	G	0.340	G	0.548
HTR2B	rs17619600	0.076	C	0.084	C	0.920
HTR2B	rs10194776	0.333	T	0.390	T	0.945
HTR2B	rs4973377	0.158	A	0.167	A	0.320
KIT	rs6554198	0.408	G	0.454	G	0.293
KIT	rs759083	0.400	G	0.341	G	0.616
KIT	rs2237025	0.392	A	0.481	A	0.800
KIT	rs13135792	0.331	C	0.325	C	0.178
KIT	rs2298976	0.125	C	0.126	C	0.510
KIT	rs11735550	0.100	C	0.133	C	0.409
KIT	rs4864920	0.217	T	0.217	T	0.482
KIT	rs1008658	0.342	A	0.354	A	0.116
KIT	rs2213180	0.193	G	0.196	G	0.938
KITLG	rs11104903	0.100	G	0.135	G	0.805
KITLG	rs11610915	0.067	A	0.012	A	0.967
KITLG	rs10858753	0.108	T	0.053	T	0.858
KITLG	rs10858758	0.192	G	0.235	G	0.467

LYST	rs6699717	0.458	T	0.493	T	0.653
LYST	rs3754230	0.150	A	0.106	A	0.778
LYST	rs11810173	0.161	T	0.170	T	0.935
LYST	rs6429238	0.333	T	0.363	T	0.538
LYST	rs3768051	0.117	T	0.126	T	0.846
LYST	rs7541057	0.475	T	0.497	C	0.860
LYST	rs17714318	0.085	A	0.073	A	0.855
MCAM	rs2511837	0.491	C	0.453	T	0.738
MCAM	rs2249466	0.258	T	0.304	T	0.633
MCAM	rs6589732	0.475	A	0.390	A	0.359
MCOLN3	rs7522239	0.136	A	0.151	A	0.930
MCOLN3	rs10782537	0.225	C	0.232	C	0.614
MCOLN3	rs10518327	0.267	A	0.275	A	0.473
MCOLN3	rs12735211	0.056	A	0.074	A	0.754
MCOLN3	rs10873682	0.492	G	0.434	A	0.718
MCOLN3	rs2304641	0.178	A	0.184	A	0.716
MCOLN3	rs6674050	0.288	C	0.202	C	0.316
MCOLN3	rs12030837	0.129	T	0.134	T	0.653
MITF	rs11128152	0.183	T	0.178	T	0.849
MITF	rs13072665	0.075	A	0.090	A	0.634
MITF	rs7623610	0.442	A	0.482	G	0.542
MITF	rs3821364	0.300	G	0.246	G	0.525
MITF	rs2131025	0.142	C	0.070	C	0.893
MLANA	rs10758717	0.250	C	0.256	C	0.595
MLANA	rs10815300	0.200	T	0.189	T	0.691
MLANA	rs2150702	0.475	C	0.472	C	0.703
MLANA	rs10975339	0.331	T	0.324	T	0.725
MLANA	rs10815303	0.112	T	0.120	T	0.957
MLANA	rs7872509	0.200	C	0.194	C	0.682
MLANA	rs10815304	0.217	C	0.229	C	0.932
MLANA	rs1056796	0.348	T	0.309	T	0.678
MLPH	rs7606177	0.233	C	0.242	C	0.648
MLPH	rs880931	0.158	T	0.119	T	0.198
MLPH	rs729389	0.142	A	0.145	A	0.480
MLPH	rs2292881	0.075	T	0.081	T	0.740
MLPH	rs13011946	0.067	T	0.044	T	0.989
MLPH	rs13383648	0.083	C	0.112	C	0.764
MLPH	rs10173589	0.217	G	0.159	G	0.969
MUTED	rs2748376	0.292	T	0.411	T	0.856
MUTED	rs3734591	0.175	C	0.183	C	0.860
MUTED	rs9328451	0.192	T	0.182	T	0.596
MUTED	rs2743989	0.275	T	0.375	T	0.499
MUTED	rs2815155	0.458	T	0.437	C	0.397

MYO7A	rs3740760	0.208	C	0.258	C	0.246
MYO7A	rs7105374	0.314	A	0.408	A	0.427
MYO7A	rs762667	0.442	C	0.332	C	0.750
MYO7A	rs11605022	0.474	G	0.425	G	0.461
MYO7A	rs3740763	0.367	A	0.406	A	0.500
MYO7A	rs948970	0.358	G	0.494	G	0.231
MYO7A	rs3758708	0.058	A	0.084	A	0.773
MYO7A	rs948962	0.383	G	0.474	T	0.534
MYO7A	rs885442	0.308	A	0.432	A	0.281
MYO7A	rs7123925	0.367	G	0.449	G	0.882
MYO7A	rs12793189	0.424	G	0.493	G	0.352
MYO7A	rs12793619	0.345	A	0.259	A	0.573
MYO7A	rs11237123	0.258	A	0.186	A	0.929
MYO7A	rs883223	0.317	A	0.337	A	0.826
NF1	rs2953016	0.271	G	0.196	G	0.904
NF1	rs2953014	0.246	C	0.230	C	0.974
NF1	rs1013948	0.233	G	0.145	G	0.585
NF1	rs10438801	0.458	G	0.356	G	0.756
NRAS	rs926938	0.408	T	0.488	C	0.919
NRAS	rs14804	0.217	T	0.216	T	0.782
NRAS	rs969273	0.317	C	0.348	C	0.077
NRAS	rs8453	0.242	A	0.115	A	0.603
PAX3	rs12620338	0.258	A	0.223	A	0.452
PAX3	rs7559271	0.375	G	0.381	G	0.296
PAX3	rs2033806	0.117	T	0.080	T	0.660
PAX3	rs2276630	0.133	T	0.099	T	0.991
PAX3	rs13405641	0.207	A	0.269	A	0.426
PAX3	rs12623857	0.167	A	0.139	A	0.864
PAX3	rs3770210	0.075	A	0.081	A	0.941
PAX3	rs16863657	0.158	G	0.124	G	0.801
PCNA	rs3729558	0.466	G	0.434	G	0.693
PCNA	rs17349	0.117	T	0.117	T	0.392
PLDN	rs16945097	0.183	T	0.135	T	0.879
PLDN	rs12909221	0.475	C	0.421	C	0.415
POMC	rs6734859	0.067	T	0.146	T	0.809
POMC	rs1866146	0.283	C	0.325	C	0.326
POMC	rs7565877	0.142	G	0.115	G	0.418
POMC	rs934778	0.358	C	0.358	C	0.057
POMC	rs874401	0.150	T	0.181	T	0.763
PRKAR1A	rs8066131	0.308	G	0.294	G	0.940
PRKAR1A	rs4968898	0.175	G	0.207	G	0.800
PRKAR1A	rs8076465	0.342	A	0.310	A	0.681
PRKAR1A	rs11651687	0.228	A	0.185	A	0.738
PRKAR1A	rs8905	0.117	G	0.163	G	0.913
PTCH1	rs16909865	0.100	G	0.073	G	0.938
PTCH1	rs574688	0.254	G	0.299	G	0.865
PTCH1	rs2297087	0.233	G	0.199	G	0.035 *
PTCH1	rs473902	0.059	C	0.108	C	0.974
PTCH1	rs4448343	0.317	G	0.288	G	0.414
PTCH2	rs3795719	0.172	G	0.132	G	0.635
PTCH2	rs7554177	0.375	A	0.291	A	0.286

RAB27A	rs1061824	0.225	G	0.179	G	0.879
RAB27A	rs9920165	0.133	G	0.103	G	0.544
RAB27A	rs16976177	0.192	G	0.144	G	0.005 **
RAB27A	rs17238192	0.133	T	0.106	T	0.758
RAB27A	rs16976194	0.308	T	0.446	T	0.328
RAB27A	rs7496857	0.289	T	0.257	T	0.940
RAB27A	rs12050885	0.167	G	0.176	G	0.893
RAB27A	rs11071175	0.467	A	0.487	G	0.678
RAB27A	rs1007912	0.075	T	0.080	T	0.489
RAB27A	rs7167572	0.308	T	0.358	T	0.185
RAB27A	rs11855084	0.092	A	0.068	A	0.718
RAB38	rs9144	0.417	C	0.407	C	0.387
RAB38	rs1027027	0.208	A	0.225	A	0.617
RAB38	rs524121	0.075	C	0.060	C	0.744
RAB38	rs12576251	0.283	G	0.341	G	0.239
RAB38	rs9666730	0.117	T	0.154	T	0.692
RAB38	rs302646	0.183	A	0.124	A	0.876
RAB38	rs12295107	0.100	A	0.072	A	0.869
RAB38	rs11602163	0.292	G	0.327	G	0.256
RABGGTA	rs941505	0.100	T	0.075	T	0.633
RABGGTA	rs3940231	0.350	A	0.401	A	0.887
RGS1	rs1359062	0.217	G	0.176	G	0.516
RGS1	rs10921202	0.092	T	0.051	T	0.831
RGS1	rs1923949	0.186	G	0.263	G	0.711
RGS1	rs2816306	0.100	C	0.109	C	0.824
RGS20	rs10958392	0.442	T	0.431	T	0.486
RGS20	rs6473895	0.108	A	0.065	A	0.975
RGS20	rs11783925	0.225	T	0.244	T	0.465
RGS20	rs1123133	0.050	G	0.032	G	0.852
RGS20	rs2220093	0.125	G	0.105	G	0.419
RGS20	rs7824575	0.283	A	0.223	A	0.886
RGS20	rs11783652	0.333	A	0.344	A	0.944
RGS20	rs6981243	0.425	C	0.452	C	0.991
RGS20	rs4738519	0.117	C	0.122	C	0.730
SILV	rs2291615	0.300	A	0.261	A	0.418
SILV	rs10783775	0.192	G	0.182	G	0.294
SILV	rs2069398	0.100	A	0.104	A	0.743
SLC45A2	rs35401	0.308	G	0.357	G	0.804
SLC45A2	rs35403	0.415	C	0.470	C	0.106
SLC45A2	rs35405	0.450	T	0.425	T	0.950
SLC45A2	rs7718382	0.246	A	0.191	A	0.787
SLC45A2	rs35414	0.308	T	0.454	T	0.012 *
SLC45A2	rs3756464	0.373	T	0.438	T	0.933
SLC45A2	rs35415	0.358	A	0.469	A	0.026 *
SNAI2	rs2735455	0.117	A	0.077	A	0.797
SNAI2	rs1992375	0.458	A	0.497	T	0.624

SNX10	rs13222190	0.475	A	0.335	A	0.662
SNX10	rs3801890	0.300	G	0.348	G	0.811
SNX10	rs1406754	0.465	T	0.359	T	0.393
SNX10	rs7782538	0.175	T	0.208	T	0.735
SNX10	rs2699808	0.400	C	0.463	C	0.799
SNX10	rs1468286	0.425	T	0.441	T	0.597
SOX10	rs139879	0.417	T	0.381	T	0.702
SOX11	rs6432221	0.325	T	0.390	T	0.115
SOX11	rs3922853	0.175	T	0.185	T	0.804
SOX11	rs17362772	0.100	G	0.108	G	0.974
SOX11	rs4371338	0.325	A	0.407	A	0.805
SOX11	rs4669779	0.383	C	0.446	C	0.790
SOX4	rs9368326	0.217	G	0.194	G	0.682
SOX4	rs1620996	0.183	T	0.130	T	0.470
SOX9	rs2229989	0.250	T	0.202	T	0.277
TYR	rs5021654	0.317	C	0.351	C	0.625
TYR	rs1042602	0.417	A	0.474	A	0.495
TYR	rs12270717	0.200	C	0.234	C	0.753
TYR	rs17793678	0.195	T	0.229	T	0.637
TYR	rs11018535	0.092	C	0.046	C	0.732
TYR	rs2186640	0.317	G	0.358	G	0.674
TYR	rs17174064	0.083	C	0.069	C	0.520
TYRP1	rs11791497	0.067	C	0.062	C	0.401
TYRP1	rs17346161	0.085	T	0.051	T	0.689
TYRP1	rs10809828	0.196	G	0.269	G	0.784
TYRP1	rs683	0.308	C	0.415	C	0.112
VPS33A	rs7388	0.292	A	0.231	A	0.853
WNT1/WNT10B	rs833820	0.425	C	0.404	C	0.134
WNT1/WNT10B	rs3782353	0.342	A	0.430	A	0.231
WNT1/WNT10B	rs833839	0.475	A	0.462	T	0.051
WNT1/WNT10B	rs7311091	0.117	T	0.084	T	0.698
WNT3A	rs697763	0.375	C	0.325	C	0.546
WNT3A	rs708122	0.371	T	0.374	T	0.570
WNT3A	rs947631	0.467	C	0.398	C	0.535
WNT3A	rs766972	0.308	G	0.296	G	0.594

HM. HapMap; SP. Spanish; MAF. Minor Allele Frequency; HWE. Hardy-Weinberg equilibrium. *p<0.05; **p<0.01; ***SNPs located in Chromosome X.
