# Supplementary material for: A Customized Pigmentation SNP Array Identifies a Novel SNP Associated with Melanoma Predisposition in the SLC45A2 Gene
Source: PLoS One. 2011 Apr 29;6(4):e19271. doi: 10.1371/journal.pone.0019271 (PMC3084811; doi:10.1371/journal.pone.0019271)
Supplement: Supplementary file 4 [file pone.0019271.s003.doc]

Table S2. List of SNPs associated with Malignant Melanoma with p-*value*<0.05.

					Controls		Cases						
**SNP_Name**	**Gene_Symbol**	**Chr**	**MaA**	**MiA**	**MaA Fq**	**MiA Fq**	**MaA Fq**	**MiA Fq**	**OR**	**CI95%**		**p-*value***	**p-Fisher**
**rs2069398**	**CDK2**	**12**	**G**	**A**	**0.90**	**0.10**	**0.94**	**0.06**	**0.52**	**0.38**	**0.72**	**0.0001 ****	**0.0001 ****
**rs35414**	**SLC45A2**	**5**	**C**	**T**	**0.55**	**0.45**	**0.63**	**0.37**	**0.71**	**0.60**	**0.84**	**0.0001 ****	**0.0001 ****
**rs35415**	**SLC45A2**	5	C	A	0.53	0.47	0.61	0.39	0.73	0.62	0.87	0.0004 **	0.0004 **
**rs3758708**	**MYO7A**	11	G	A	0.92	0.08	0.87	0.13	1.63	1.20	2.22	0.0016 **	0.0019 **
**rs1510521**	**ADAMTS20**	12	T	C	0.69	0.31	0.63	0.37	1.30	1.09	1.55	0.0039 **	0.0044 **
**rs17793678**	**TYR**	11	C	T	0.77	0.23	0.72	0.28	1.32	1.09	1.60	0.0049 **	0.0052 **
**rs13135792**	**KIT**	4	T	C	0.67	0.33	0.62	0.38	1.27	1.07	1.52	0.0071 **	0.0081 **
**rs35405**	**SLC45A2**	5	C	T	0.58	0.42	0.52	0.48	1.26	1.06	1.49	0.0084 **	0.0086 **
**rs759083**	**KIT**	4	C	G	0.66	0.34	0.6	0.4	1.26	1.06	1.50	0.0090 **	0.0099 **
**rs948970**	**MYO7A**	11	C	G	0.51	0.49	0.56	0.44	0.80	0.68	0.94	0.0098 **	0.0099 **
**rs880931**	**MLPH**	2	C	T	0.88	0.12	0.91	0.09	0.70	0.53	0.92	0.0110 *	0.0126 *
**rs6981243**	**RGS20**	8	A	C	0.55	0.45	0.60	0.40	0.80	0.68	0.95	0.0113 *	0.0118 *
**rs2062731**	**ADAMTS20**	12	A	G	0.89	0.11	0.92	0.08	0.69	0.51	0.92	0.0118 *	0.0137 *
**rs12270717**	**TYR**	11	T	C	0.77	0.23	0.72	0.28	1.28	1.05	1.55	0.0125 *	0.0127 *
**rs17093450**	**ADAMTS20**	12	G	A	0.86	0.14	0.90	0.10	0.72	0.56	0.93	0.0128 *	0.0148 *
**rs6554198**	**KIT**	4	A	G	0.55	0.45	0.60	0.40	0.81	0.68	0.95	0.0130 *	0.0134 *
**rs4987852**	**BCL2**	18	A	G	0.95	0.05	0.92	0.08	1.52	1.08	2.15	0.0157 *	0.0173 *
**rs8059139**	**CDH1**	16	A	G	0.95	0.05	0.93	0.07	1.51	1.05	2.17	0.0246 *	0.0251 *
**rs12620338**	**PAX3**	2	G	A	0.78	0.22	0.81	0.19	0.79	0.65	0.98	0.0311 *	0.0324 *
**rs819133**	**ASIP**	20	G	T	0.86	0.14	0.89	0.11	0.76	0.59	0.98	0.0318 *	0.0331 *
**rs2237025**	**KIT**	4	G	A	0.52	0.48	0.56	0.44	0.83	0.70	0.99	0.0353 *	0.0390 *
**rs4669779**	**SOX11**	2	T	C	0.55	0.45	0.60	0.40	0.83	0.70	0.99	0.0365 *	0.0373 *
**rs2276329**	**CDH1**	16	A	G	0.96	0.04	0.94	0.06	1.48	1.01	2.16	0.0408 *	0.0485 *
**rs275630**	**ADAMTS20**	12	G	A	0.72	0.28	0.68	0.32	1.21	1.01	1.45	0.0423 *	0.0450 *
**rs11742673**	**AP3B1**	5	G	A	0.59	0.41	0.55	0.46	1.19	1.01	1.41	0.0425 *	0.0466 *
**rs729389**	**MLPH**	2	G	A	0.86	0.14	0.88	0.12	0.77	0.60	0.99	0.0425 *	0.0472 *
**rs6674050**	**MCOLN3**	1	T	C	0.80	0.20	0.76	0.24	1.23	1.01	1.51	0.0435 *	0.0443 *
**rs2511837**	**CBL**	11	C	T	0.55	0.45	0.50	0.50	1.19	1.00	1.41	0.0440 *	0.0485 *
**rs5021654**	**TYR**	11	G	C	0.65	0.35	0.61	0.39	1.19	1.00	1.42	0.0468 *	0.0511
**rs7075080**	**HPS1**	10	A	G	0.57	0.43	0.61	0.39	0.84	0.71	1.00	0.0494 *	0.0497 *

MaA. Mayor Allele; MiA. Minor Allele; Fq. Frequency; OR. Odds Ratio; CI. Confidence interval
*p<0.05; **p<0.01; Bold indicates significant results when adjusted by Bonferroni.
