# Supplementary material for: A Customized Pigmentation SNP Array Identifies a Novel SNP Associated with Melanoma Predisposition in the SLC45A2 Gene
Source: PLoS One. 2011 Apr 29;6(4):e19271. doi: 10.1371/journal.pone.0019271 (PMC3084811; doi:10.1371/journal.pone.0019271)
Supplement: Supplementary file 5 [file pone.0019271.s004.doc]

Table S3. Association between top-ten SNPs and phenotypic characteristics.

Gene	SNPs		Light Eye Colour		Fair/Pale Skin Colour		Blond/Red Hair Colour	
	rs#	mA	OR (95% CI)	p-*value*	OR (95% CI)	p-*value*	OR (95% CI)	p-*value*
KIT	rs759083	G	1.00(0.82-1.22)	0.979	1.03(0.85-1.24)	0.789	**1.48(1.15-1.90)**	**0.002 ****
	rs13135792	C	1.02(0.83-1.24)	0.862	1.07(0.89-1.30)	0.457	**1.41(1.10-1.82)**	**0.007 ****
SLC45A2	rs35405	T	1.08(0.89-1.31)	0.440	1.04(0.86-1.24)	0.710	1.20(0.93-1.53)	0.160
	rs35414	T	0.90(0.74-1.10)	0.300	**0.81(0.68-0.98)**	**0.028 ***	**0.73(0.57-0.95)**	**0.018 ***
	rs35415	A	0.94(0.78-1.14)	0.539	**0.83(0.69-1.00)**	**0.048 ***	0.78(0.60-1.00)	0.051
MYO7A	rs948970	G	0.89(0.74-1.09)	0.251	0.97(0.81-1.16)	0.770	**0.77(0.60-0.99)**	**0.041 ***
	rs3758708	A	0.99(0.71-1.37)	0.940	0.99(0.72-1.35)	0.958	1.26(0.84-1.89)	0.258
TYR	rs17793678	T	**1.28(1.03-1.59)**	**0.024 ***	1.16(0.94-1.42)	0.174	1.00(0.82-1.44)	0.546
ADAMTS20	rs1510521	C	**1.24(1.02-1.52)**	**0.034 ***	1.01(0.84-1.23)	0.899	**1.32(1.02-1.70)**	**0.035 ***
SILV-CDK2	rs2069398	A	0.83(0.58-1.19)	0.305	1.01(0.73-1.40)	0.955	0.78(0.48-1.27)	0.323

mA:Minor Allele; OR: Odds Ratio per minor allele; CI: Confidence Interval

*p<0.05; **p<0.01; Bold indicates statistically significant results.

Gene	SNPs		Childhood sunburn		Nº of Nevi ≥50		Lentigines	
	rs#	mA	OR (95% CI)	p-*value*	OR (95% CI)	p-*value*	OR (95% CI)	p-*value*
KIT	rs759083	G	**1.28(1.06-1.55)**	**0.011 ***	0.98(0.79-1.23)	0.892	0.98(0.80-1.22)	0.885
	rs13135792	C	**1.27(1.04-1.54)**	**0.017 ***	1.09(0.87-1.36)	0.458	1.00(0.81-1.23)	0.967
SLC45A2	rs35405	T	1.12(0.93-1.35)	0.236	0.96(0.77-1.19)	0.694	1.14(0.93-1.40)	0.196
	rs35414	T	0.91(0.75-1.10)	0.324	0.89(0.72-1.12)	0.344	1.07(0.87-1.31)	0.529
	rs35415	A	1.04(0.86-1.25)	0.686	0.96(0.78-1.19)	0.734	1.06(0.86-1.30)	0.583
MYO7A	rs948970	G	0.84(0.69-1.01)	0.059	0.91(0.74-1.14)	0.416	0.84(0.69-1.03)	0.102
	rs3758708	A	**1.38(1.00-1.91)**	**0.047 ***	0.95(0.65-1.37)	0.771	1.06(0.75-1.49)	0.754
TYR	rs17793678	T	1.07(0.86-1.32)	0.549	1.20(0.95-1.54)	0.132	1.03(0.82-1.29)	0.831
ADAMTS20	rs1510521	C	1.06(0.87-1.29)	0.567	**0.78(0.61-0.98)**	**0.034 ***	1.06(0.85-1.31)	0.605
SILV-CDK2	rs2069398	A	**0.69(0.49-0.98)**	**0.035 ***	0.78(0.52-1.19)	0.244	0.72(0.49-1.05)	0.089

mA:Minor Allele; OR: Odds Ratio per minor allele; CI: Confidence Interval

*p<0.05; **p<0.01; Bold indicates statistically significant results.
