# Supplementary material for: A Customized Pigmentation SNP Array Identifies a Novel SNP Associated with Melanoma Predisposition in the SLC45A2 Gene
Source: PLoS One. 2011 Apr 29;6(4):e19271. doi: 10.1371/journal.pone.0019271 (PMC3084811; doi:10.1371/journal.pone.0019271)
Supplement: Supplementary file 6 [file pone.0019271.s006.doc]

Table S5. Classification of the Spanish samples (phase I) by age and phenotype.

	Discovery Group (Phase I)		
**Characteristic**	**Cases (N=590) n (%)**	**Controls (N=507) n (%)**	**p-*value*******
**Age at diagnosis (years)**			-
Mean (SD)	52.60 (16.07)	41.62 (19.28)	
< Mean	215 (36.44)	246 (48.52)	
> Mean	210 (35.59)	231 (45.56)	
Unknown	165 (27.97)	30 (5.92)	
**Sex**			0.62
Male	234 (33.66)	224 (44.18)	
Female	316 (53.56)	283 (55.82)	
Unknown	40 (6.78)	0 (0.00)	
**Eye Colour**			**0.0002**
Light Eye Colour	177 (30.00)	140 (27.61)	
Dark Eye Colour	274 (46.44)	364 (71.79)	
Unknown	139 (23.56)	3 (0.59)	
**Hair Colour**			**2.77×10^{-14}**
Blond/Red	115 (19.49)	34 (6.71)	
Brown/Black	336 (56.95)	472 (93.1)	
Unknown	139 (23.56)	1 (0.20)	
**Skin Colour**			**0.01**
Fair Skin Colour	266 (45.08)	254 (50.1)	
Dark Skin Colour	185 (31.36)	247 (48.72)	
Unknown	139 (23.56)	6 (1.18)	
**Nº of Nevi**			**1.75×10^{-13}**
<50	296 (50.17)	402 (79.29)	
≥50	155 (26.27)	64 (12.62)	
Unknown	139 (23.56)	41 (8.09)	
**Lentigines**			**7.51×10^{-11}**
No	156 (26.44)	186 (36.69)	
Yes	293 (49.66)	132 (26.04)	
Unknown	141 (23.90)	189 (37.28)	
**Childhood sunburn**			**2.94×10^{-14}**
No	155 (26.27)	301 (59.37)	
Yes	283 (47.97)	163 (32.15)	
Unknown	152 (25.76)	43 (8.48)	
**Fitzpatrick phototype**			**7.54×10^{-10}**
I-II	200 (33.90)	48 (9.47)	
III-IV	194 (32.88)	148 (29.19)	
Unknown	196 (33.22)	311 (61.34)	

* Fisher's exact test. p-*value* excluding unknown values.
SD, estándar deviation
