# Supplementary material for: A Customized Pigmentation SNP Array Identifies a Novel SNP Associated with Melanoma Predisposition in the SLC45A2 Gene
Source: PLoS One. 2011 Apr 29;6(4):e19271. doi: 10.1371/journal.pone.0019271 (PMC3084811; doi:10.1371/journal.pone.0019271)
Supplement: Supplementary file 7 [file pone.0019271.s007.doc]

Table S6. General information about genes, chromosomal location, SNPs per gene, associated disease and function in pigmentation.

Melanocyte Development

Gene	*Genes in the genomic region*	*Chr.*	*Nº SNPs*	*Associated Disease*	*Protein Encoded*	*Function in Pigmentation*
ADAM17		*2*	5	Unknown	Disintegrin and metalloproteinase domain 17	Protease, processing various surface proteins.Irregular pigmentation in hairs
ADAMTS20		*12*	11	Unknown	Disintegrin and metalloprotease domain (reprolysin type)	Metalloprotease. Melanoblast migration? Lumbar white belt
ASIP	*AHCY*	*20*	2	Unknown	Agouti signal protein	Eumelanin / pheomelanin switch
EDNRB		*13*	5	Hirschprung disease 2	Endothelin receptor B	White spotting
GNA11		*19*	10	Unknown	Guanine nucleotide binding protein subunit Ga11	GPCR signalling: limits melanocyte proliferation. Dark skin
GNAQ		*9*	15	Diminished platelet aggregation	Guanine nucleotide binding protein subunit Gaq	GPCR signalling: limits melanocyte proliferation. Dark skin
GNAS		*20*	14	McCune-Albright syndrome, among others	Guanine nucleotide-binding protein	Hormonal regulation of adenylate cyclase
GPR143		*X*	7	Ocular Albinism 1	G-protein-coupled receptor (GPR143)	Melanosome Biogenesis and signal transduction
KIT		*4*	10	Piebaldism	(Oncogene) Receptor for Kit ligand/SCF	Required for melanoblast survival, homing. White spotting
KITLG		*12*	4	Unknown	Stem cell factor (SCF) (Kit ligand)	Melanoblast growth and differentiation factor. White spotting
MCAM	*CBL*	*11*	4	Unknown	Melanoma cell adhesion molecule	Cell adhesion, and in cohesion of the endothelial monolayer at intercellular junctions in vascular tissue
MCOLN3		*1*	9	Unknown	Cation channel	Ion homeostasis. Patches of normal, diluted and white hair.
MITF		*3*	5	Waardenburg syndrome 2	Transcription factor, master regulator of melanocyte lineage	Melanocyte differentiation. White spotting
NF1		*17*	5	Neurofibromatosis 1	RAS GTPase-activating protein Neurofibromin 1	Negative regulator of the ras signal transduction pathway. Unpigmented eyes
PAX3		*2*	8	Waardenburg syndrome 1, 3	PAX-3 transcription factor	Neural tube development
PTCH1		*9*	5	Basal cell nevus syndrome, holoprosencephaly	Patched homolog 1	Formation of embryonic structures, and tumor supressor
PTCH2		*1*	2	Nevoid basal cell carcinoma syndrome	Patched homolog 2	Tumor supressor, may have a role in epidermal development
POMC		*2*	5	Unknown	Proopiomelanocortin, including Melanocyte Stimulating Hormone	Eumelanin / pheomelanin switch (and endocrine functions)

SNAI2		*8*	2	Waardenburg syndrome 2	Snail family of C2H2-type zinc finger transcription factors	Transcriptional repressor, involved in the generation and migration of neural crest cells
SNX10		*7*	6	Unknown	Sorting nexin family 10	Intracellular trafficking
SOX11		*2*	5	Unknown	SRY (sex determining region Y)-box 11	Regulation of embryonic development and in the determination of the cell fate, probably important in the developing nervous system
SOX4		*6*	2	Unknown	SRY (sex determining region Y)-box 4	Apoptosis pathway leading to cell death as well as to tumorigenesis
SOX9		*17*	2	Campomelic dysplasia	SRY (sex determining region Y)-box 9	Skeletal development and chondrocyte differentiation
SOX10		*22*	1	Waardenburg syndrome	SRY (sex determining region Y)-box 10	White spotting
WNT1/ WNT10B	*ARF3*	*12*	4	Unknown	Wingless-type MMTV integration site family, member 10B	Probable developmental protein, may be a signaling molecule which affects the development of discrete regions of tissues
WNT3A		*1*	4	Defects of neural crest	Growth factor/morphogen,	Cell-cell signaling during morphogenesis

Melanosome contruction, transport and protein routing

Gene	*Genes in the genomic region*	*Chr.*	*Nº SNF*	*Associated Disease*	*Protein Encoded*	*Function in Pigmentation*
AP3D1	*DOT1L*	*19*	6	Unknown	Delta subunit of adaptor protein 3 complex	Organellar protein routing
AP3M2	*PLAT*	*8*	4	Unknown	Adaptor-related protein comlex 3	Trafficking to lysosomes and specialized organelles
BLOC1S3	*TRAPPC6A*	*19*	1	Hermansky Pudlak 8	Unknown	Component of the BLOC1 protein transport complex
CNO		*4*	3	Unknown	Unknown	Organelle biogenesis
DCT		*13*	6	Unknown	DOPAchrome tautomerase, melanosomal enzyme	Dilution of eumelanin color
HPS1		*10*	10	Hermansky Pudlak 1	Membrane protein	Organelle biogenesis and size
HPS2		*5*	14	Hermansky Pudlak 2	Beta-3 subunit of adaptor protein 3 complex	Organellar protein routing
HPS4		*22*	7	Hermansky Pudlak 4	Unknown	Organelle biogenesis and size
HPS5		*11*	6	Hermansky Pudlak 5	Unknown	Biogenesis of lysosome-related organelles complex-2
HPS6		*10*	3	Hermansky Pudlak 6	Unknown	Biogenesis of lysosome-related organelles complex-2
LYST		*1*	7	Chediak Higashi	Membrane protein	Organelle biogenesis and size

MLANA		*9*	8	Unknown	Melan-A	Expression, stability, trafficking, and processing of melanocyte protein SILV/PMEL17, critical to the formation of stage II melanosomes
MLPH		*2*	9	Griscelli syndrome	Melanophilin	Melanosome transport
MUTED		*6*	6	Unknown	BLOC-1 subunit, TXNDC5	Organelle biogenesis
MYO7A		*11*	15	Usher syndrome 1B	Myosin type VIIa	Melanosome transport
PLDN		*15*	2	Unknown	Pallidin	Vesicle-docking and fusion
RAB27A		*15*	11	Griscelli syndrome	RAS associated protein	Melanosome transport
RAB38		*11*	8	Unknown	Member of RAS oncogene family	Targeting of Tyrp1 protein to the melanosome
RABGGTA		*14*	2	Unknown	Alpha-subunit of rab geranylgeranyl transferase	Organelle biogenesis
SILV	*CDK2, DGKA*	*12*	3	Unknown	Melanosomal matrix protein	Trapping of melanin intermediates
SLC45A2		*5*	7	OCA 4	Solute transporter, Membrane-associated transporter protein	Severe dilution coat and eye pigment
TYR		*11*	8	OCA 1	Melanogenic enzyme	No pigment in null mice
TYRP1		*9*	4	OCA 3	Melanosomal enzyme/stabilizing factor	Brown eumelanin
VPS33A		*12*	1	Unknown	Vacuolar protein sorting 33a	Organellar protein routing

Systemic effects, non pigmentation

Gene	***Genes in the genomic region***	***Chr.***	***Nº SNF***	***Associated Disease***	***Protein Encoded***	***Function in Pigmentation***
BCL2		*18*	6	Follicular lymphomas	Integral outer mitochondrial membrane protein	Inhibitor of apoptosis
BCL2A1		*15*	4	Unknown	BCL-2 protein family 1A	Reduce the release of pro-apoptotic cytochrome c from mitochondria and block caspase activation
CDH1		*16*	9	Unknown	Calcium dependent cell-cell adhesion glycoprotein	Progression in cancer by increasing proliferation, invasion, and/or metastasis
CDH3		*16*	7	Congential hypotrichosis with juvenile macular dystrophy	Calcium dependent cell-cell adhesion glycoprotein	Connecting cells and sorting of heterogeneous cell types
CDK4		*12*	3	Melanoma and other cancers	Serine/threonine protein kinase 4	Controls cell cycle G1 progression
CDKN2A		*9*	4	Familial melanoma and other cancers	Cyclin-dependent kinase inhibitor 2A	Controls cell cycle G1 control, tumor suppressor
CDKN2B		*9*	5	Melanoma and other cancers	Cyclin-dependent kinase inhibitor 2B	Controls cell cycle G1 progression
CTNNBIP1	*LZIC*	*1*	5	Unknown	Beta-catenin-interacting protein 1	Negative regulator of the Wnt signaling pathway

F2RL1		*5*	6	Unknown	Coagulation factor II (thrombin) receptor-like 1	May have a role in the regulation of vascular tone
HRK		*12*	4	Unknown	Harakiri, BCL2 interacting protein	Activator of apoptosis
HTR2B	*PSMD1*	*2*	4	Unknown	Serotonin receptor 2B	Regulation of differentiation and proliferation of the embryonic and adult heart, gastrointestinal contraction and endothelium-dependent relaxation
NRAS	*CSDE1*	*1*	4	Juvenile myelomonocytic leukemia	Neuroblastoma RAS viral oncogene homolog	Oncogene, intrinsic GTPase activity
PCNA		*20*	2	Unknown	Proliferating cell nuclear antigen	Control of eukaryotic DNA replication
PRKAR1A		*17*	5	Carney complex	Protein kinase, cAMP-dependent, regulatory, type I, alpha	Regulation of lipid and glucose metabolism,component of the signal transduction mechanism of certain GPCRs
RGS1		*1*	4	Unknown	Regulator of G-protein signaling 1	Inhibits signal transduction, may be involved in the regulation of B-cell activation and proliferation
RGS20		*8*	9	Unknown	Regulator of G-protein signaling 20	Inhibits signal transduction
Total			384			
